# Incidence and predictors of second-line antiretroviral treatment failure among adults living with HIV in Amhara region: a multi-centered retrospective follow-up study

**DOI:** 10.1186/s12879-019-4243-5

**Published:** 2019-07-09

**Authors:** Muluneh Alene, Tadesse Awoke, Melaku Kindie Yenit, Adino Tesfahun Tsegaye

**Affiliations:** 1 Department of Public Health, Debre Markos University, Debre Markos, Ethiopia; 2 Department of Epidemiology and Biostatistics, University of Gondar, Gondar, Ethiopia

**Keywords:** Predictors, Second-line, ART, Adults, Amhara region

## Abstract

**Background:**

Second-line Antiretroviral Therapy (ART) regimens are used when patients develop treatment failure for first-line drug regimens. It is costly unaffordable and it is not widely available for patients in resource limiting setting, there is a need to maximizing the duration of stay on second-line regimen. This study was conducted to estimate the incidence rate of second-line treatment failure and to identify its predictors among adults living with HIV in the Amhara region.

**Methods:**

An institution based retrospective follow-up study was conducted from May to June 2017. A total of 1,011 adults on second-line ART who were enrolled between February 2008 and April 2016 were included for final analysis. Kaplan-Meier estimator curves were used to describe the survival function. Semi-parametric proportional hazard model was fitted to identify the predictors of treatment failure.

**Results:**

The overall incidence of second-line treatment failure was 9.86 per 100 person-years. It was high during the first and the last year of follow-up. The rate of second-line treatment failure was higher for patients who didn’t change second-line regimens (HR: 1.55, 95%CI: 1.18–2.04), who had poor ART adherence (HR: 1.40, 95%CI: 1.06–1.85), and not taking INH (HR: 1.68, 95%CI: 1.23–2.30) as compared to their counter group. The rate of treatment failure for patients who were under WHO clinical stage III at switch (HR: 0.68, 95%CI: 0.50–0.91) was also lower as compared to clients who were under WHO clinical stage I. Furthermore, the rate of treatment failure was higher for clients who were under second-line regimen “TDF-3TC-LPV/r” (HR: 1.55, 95%CI: 1.03–2.32) and “AZT-3TC-LPV/r” (HR: 3.00, 95%CI: 1.86–4.85) as compared to patients under “ABC-ddI-LPV/r” regimens.

**Conclusions:**

A high incidence rate of second-line treatment failure was noticed in the study setting. The rate of second-line treatment failure was higher for patients who didn’t change drug regimens, who had poor ART adherence, and who were not taking INH. Therefore, addressing significant predictors to prevent treatment failure among ART patients is essential and sustainable monitoring to reduce the risk of treatment failure is also desirable.

## Background

The Human Immunodeficiency Virus (HIV) has continued to be a major public health problem and claimed more than 39 million deaths since the beginning of the pandemic [1]. Globally, approximately 36.7 million people were living with HIV at the end of 2016, including 1.8 million people who were newly infected [2]. Cognizant of the magnitude of the problem, the World Health Organization (WHO) has recommended the initiation of ART for people living with HIV. ART is essential for the restoration of immunological function, suppression of viral replication, and improvement of the quality of life [3–5]. It is expanding rapidly, and about 19.5 million people living with HIV were receiving ART in 2016, and AIDS-related deaths are fell by one-third, and the infection has been transformed into a manageable chronic condition [4, 6].

Optimal adherence to ART will minimize the development of drug resistance to Antiretroviral (ARV) medication, and it is key to suppress viral load, and decreasing the risk of mortality [7–9]. Despite, ART reduces HIV associated morbidity and mortality, non-adherence, drug resistance, and treatment failure are creating a significant challenge to achieving better treatment response. Antiretroviral treatment failure, which could be clinical, immunological or virological, occurs when the ART regimen is unable to control viral replication [6, 10]. Routine viral load monitoring was not available during the follow-up period of the current study, and response to treatment was assessed by CD4 cell count and clinical parameters [4, 6, 10]. For most patients on treatment, the adequate immunologic response is defined as an increase in CD4 count in the range of 50–150 cells/mm^3^ during the first years of ART follow-up and an average increase from 50 to 100 cells/mm^3^ per year at a steady state after a year [11, 12].

Second-line ART regimens are used when patients develop treatment failure for the first-line treatment regimens. It is estimated that 18.8% of people living with HIV were experienced second-line treatment failure in low-income countries [13, 14]. In Ethiopia, about 344,344 people were using ART in 2014, and out of them 1.5% were on second-line treatment. A study conducted in northwest Ethiopia indicated that immunological failure was high, where 21% of patients had developed treatment failure [15]. Previous reports showed that patients CD4 cell count at the switch, WHO clinical stage at the switch, functional status at the switch, TB co-infected and ART adherence were significant covariates of treatment failure [13, 16, 17].

Second-line treatment is costly unaffordable, and it is not widely available for patients in resource limiting setting. There is a need to maximizing the duration of stay on the second-line regimen. Thus, this study was conducted to estimate the incidence rate of second-line treatment failure and to identify its predictors among adults living with HIV in the Amhara Region. The study has both public health and clinical importance. The suppression of viral load and reduction of the risk of treatment failure decreases HIV transmission, increases productivity, and enables to maximize the duration on second-line treatment.

## Methods

### Study design and setting

A multi-centered institution based retrospective follow-up study was conducted from May to June 2017. The Zonal hospitals of Amhara region namely, University of Gondar, Felege Hiwot referral, Debre Markos, Dessie referral, Debre Tabor, Woldiya, Finote Selam, and Debre Berhan hospitals were included in this study. All adults aged 15 and above and started second-line treatment between February 2008 and April 2016 were the study population. Accordingly, all adults aged 15 and above who were on second-line ART follow-up between February 2008 and April 2016 in the selected hospitals were included in the study. This study excludes HIV positive individuals with incomplete baseline information’s of CD4 cell count and WHO clinical stage.

To get a representative image for the source population, the optimal sample size was calculated using sample size determination formula for survival analysis with assumptions of 95% level of confidence and 80% power, and it was 1098. However, the total numbers of patients on second-line treatment who fulfill the eligibility criteria in eight hospitals were 1,011, and all of these were included for the final analysis.

### Outcome and predictor variables

The outcome variable of this study was time to second-line treatment failure. In this study, antiretroviral treatment failure is defined as a clinical failure, an immunological failure, or both. Clinical failure for adults is defined as a new or recurrent clinical event indicating severe immunodeficiency (WHO clinical stage 4 condition and certain WHO clinical stage 3 conditions (pulmonary TB and severe bacterial infections)) after 6 months of effective treatment. On the other hand, immunological failure is declared, when patients have a CD4 count at or below 250 cells/mm^3^ following clinical failure or persistent CD4 levels below 100 cells/mm^3^ (at least two consecutive CD4 cell count in a row is less than100 cells/mm^3^) [6]. Patients on second-line ART who were lost-to-follow-up, transferred-out, and died were considered as censored. For this study, lost-to-follow-up refers clients stopped ART follow-up for 3 months or longer due to different reasons. Though patient’s death and lost-to-follow-up may associate with treatment failure, we didn’t have confirmation whether these patients actually died, or lost follow-up due to treatment failure. Therefore, in this study, death, and lost-follow-up were considered as censored. Patients who didn’t experience treatment failure criteria in the above-mentioned follow-up period were also considered as censored.

Socio-demographic variables (age, sex, educational status, and functional status at switch), body mass index (BMI) at switch, taking Isoniazid (INH) preventive therapy, opportunistic infections (OI), CD4 cell count at switch, regimen modifications, WHO clinical stage (I-IV) at switch and ART adherence were analyzed in this study. ART adherence was measured by considering patients clinical compliance in scheduled visits. Patients adherence was classified as poor, fair, and good if there level of adherence was < 85, 85–95%, and > 95% respectively. Regimen modification was also considered when patients had regimen modification at least once in the follow-up period.

### Data collection tool and procedures

The data collection checklist was organized to abstract data from the national ART follow-up form. This checklist was prepared for the collection of both baseline and follow-up socio-demographic, clinical, immunological, and treatment outcome related information that are important for the assessment of treatment failure. Consequently, the data were collected through chart review by trained nurses.

All records of HIV patients who were switched to second-line ART between February 2008 and April 2016 were considered. Charts were retrieved using patient medical record numbers and ART registration numbers found in the database of health facilities. To assess the immunological and clinical response of the treatment, this study includes patients who had records of at least two CD4 cell count measurement and WHO-clinical stage. The quality of data was also assured by using a pretested checklist and trained data collectors. Data completeness and consistency were also checked by supervisors on daily bases. Moreover, the data clerks and case managers assisted data collectors by identifying patient records.

### Data processing and analysis

The extracted data were checked for completeness, coded, entered, and cleaned into EPI-INFO version 7 and exported to R version 3.4 software for further analysis. An incidence rate was computed using person-time of observations. Person-time is the sum of the amount of time contributed by study participants in the follow-up period. Kaplan-Meier estimator curves were used to describe the survival function and to estimate the median survival time. Semi-parametric proportional hazard model was also performed to identify the predictors of second-line treatment failure. In semi-parametric model the baseline hazard function doesn’t need to be follow a particular statistical distribution which makes more robust than parametric approaches because it is not vulnerable to misspecification of the baseline hazard.

The proportional hazard assumption was checked by Schoenfeld residuals (Table 1). This assumption is supported by anon-significant association between residuals and time. Variables which have a 95% confidence interval for hazard ratio (HR) without including one were considered as to be significant predictors of second-line treatment failure.

**Table 1:** Test of proportional hazard (PH) assumption using Schoenfeld residuals from second-line ART among adults in Amhara Region (February 2008–April 2016)

Variables	Correlation	Chi-square	*P*-value
Age			
15–24	Reference		
25–34	−0.016	0.069	0.791
35–44	−0.004	0.004	0.947
≥ 45	− 0.055	0.805	0.369
Gender			
Female	Reference		
male	0.011	0.032	0.857
BMI(kg/m^2^)			
< 18.5	−0.106	2.900	0.089
18.5–24.99	Reference		
> 25	0.037	0.327	0.567
CD4 count at switch			
≥ 100 cells/mm^3^	Reference		
< 100 cells/mm^3^	−0.089	2.098	0.147
Regimen modification			
Yes	Reference		
No	0.002	0.001	0.973
ART adherence			
Good	Reference		
Fair	0.066	1.132	0.287
Poor	0.079	1.612	0.204
WHO clinical stage			
I	Reference		
II	0.045	0.519	0.471
III	0.052	0.728	0.393
IV	0.079	1.679	0.195
Isoniazid Preventive Therapy **(**INH) given			
Yes	Reference		
No	0.013	0.044	0.833
Functional status at switch			
Working	Reference		
Ambulatory	−0.056	0.867	0.352
bedridden	−0.023	0.128	0.719
Opportunistic Infections (OI)			
Yes	Reference		
No	0.093	2.290	0.130
Second-line regimen			
ABC-ddI-LPV/r	Reference		
TDF-3TC-LPV/r	−0.058	0.906	0.341
AZT-3TC-LPV/r	0.017	0.072	0.788
Others^a^	−0.077	1.512	0.219

^a^ *= (ABC-ddI-NFV, AZT-3TC-ATV/r, TDF-3TC-ATV/r, ABC-3TC-LPV/r, and ABC-3TC-ATV/r)*

## Results

### Characteristics of second-line ART clients

A total of 1,011 records of HIV positive patients on second-line treatment with complete information were analyzed, while 222 records of patients were excluded from the analysis because CD4 cell count and WHO-clinical stage didn’t measured for a minimum of two times. Nearly half (50.74%) of the subjects were male and about two-thirds (64.47%) were below the age of 35 years. About two-thirds (60.44%) of the ART clients started second-line ART at CD4 cell count level of below 100 cells/mm^3^.

Second-line regimens were modified for 576 (56.97%) patients. Nearly one-third (34.92%) of patients had poor ART adherence, 734 (72.60%) didn’t take INH preventive therapy, and 78 (7.72%) of patients had at least one opportunistic infection. Nearly half (46.3%), 8.8, 36.7, and 8.2% of patients were started second-line treatment under WHO clinical stage-I, II, III and IV respectively. The majority (84.67%) of clients were initiated second-line treatment on working functional status. About 39.07% of the HIV/AIDS patients were underweight (below 18.5 kg/m^2^ of BMI). Among ART clients who experienced second-line treatment failure, about 43.7, 46.46, and 83.74% of them were underweight, females, and had poor ART adherence respectively. Similarly, among clients who experienced treatment failure, nearly 56 and 45% of them were started second line ART at CD4 cell count level of below 100 cells/mm^3^ and under WHO clinical stage I respectively (Table 2).

**Table 2:** Descriptive results of socio demographic, treatment related and clinical characteristics of patients on second-line treatment in Amhara region (February 2008 – April 2016)

Variable	Total N (%)	Treatment failure	
		Failure N (%)	Censored N (%)
Age			
15–24	105 (10.39)	28 (11.02)	77 (10.17)
25–34	490 (54.08)	124 (48.82)	366 (48.35)
35–44	299 (32.71)	74(29.13)	225 (29.72)
≥ 45	117(10.37)	28(11.02)	89 (11.76)
Gender			
Female	498 (49.26)	118 (46.46)	380 (50.20)
Male	513 (50.74)	136 (53.54)	377 (49.80)
BMI(kg/m^2^)			
< 18.5	395 (39.07)	111 (43.70)	409 (54.03)
18.5–24.99	538 (53.21)	129 (50.79)	284 (37.52)
> 25	78 (7.72)	14 (5.51)	64 (8.45)
CD4 count at switch			
≥ 100 cells/mm^3^	400 (39.56)	111(43.70)	289 (38.18)
< 100 cells/mm^3^	611(60.44)	143 (56.30)	468 (61.82)
Regimen modification			
Yes	435 (43.03)	85 (33.46)	350(46.24)
No	576 (56.97)	169 (66.54)	407(53.76)
ART adherence			
Good	462 (45.70)	101 (39.76)	361(47.69)
Fair	196 (19.39)	43 (16.93)	153 (20.21)
Poor	353 (34.92)	110 (43.31)	243 (32.10)
WHO clinical stage			
I	468 (46.29)	114 (44.88)	354 (46.76)
II	89 (8.80)	20 (7.87)	69 (9.11)
III	371 (36.70)	84 (33.07)	287(37.91)
IV	83 (8.21)	36 (14.17)	47 (6.21)
INH given			
Yes	277 (27.40)	51(20.08)	226 (29.85)
No	734 (72.60)	203 (79.92)	531(70.15)
Functional status at switch			
Working	856 (84.67)	206 (83.74)	650 (85.87)
Ambulatory	137 (13.55)	40 (16.26)	97 (12.81)
bedridden	18 (1.78)	8 (3.25)	10 (1.32)
Opportunistic Infections (OI)			
Yes	78 (7.72)	18 (7.09)	60 (7.93)
No	933 (92.28)	236 (92.91)	697 (92.07)
Second-line regimen			
ABC-ddI-LPV/r	189 (18.69)	35 (13.78)	154 (20.34)
TDF-3TC-LPV/r	351 (34.72)	94 (37.01)	257(33.95)
AZT-3TC-LPV/r	106 (10.48)	37 (14.57)	69 (9.11)
Others^a^	365 (36.10)	88 (34.65)	277(36.59)

^a^ *= (ABC-ddI-NFV, AZT-3TC-ATV/r, TDF-3TC-ATV/r, ABC-3TC-LPV/r, and ABC-3TC-ATV/r)*

**Fig. 1:**
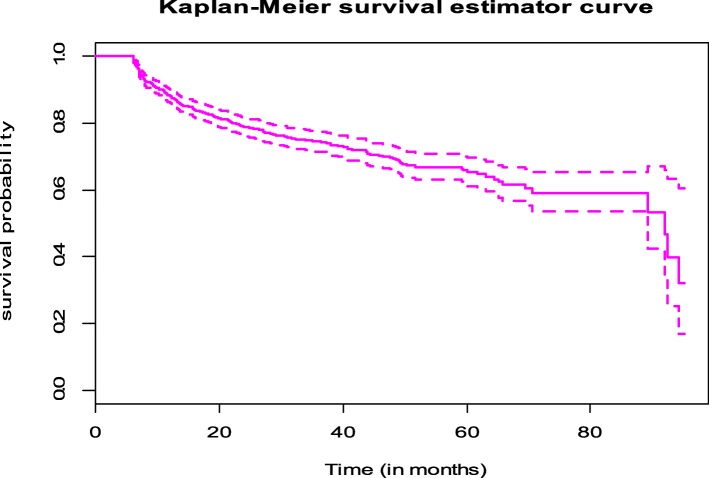
Kaplan-Meier survival estimator curve of second-line ART failure among adults in Amhara region (February 2008 – April 2016)

**Fig. 2:**
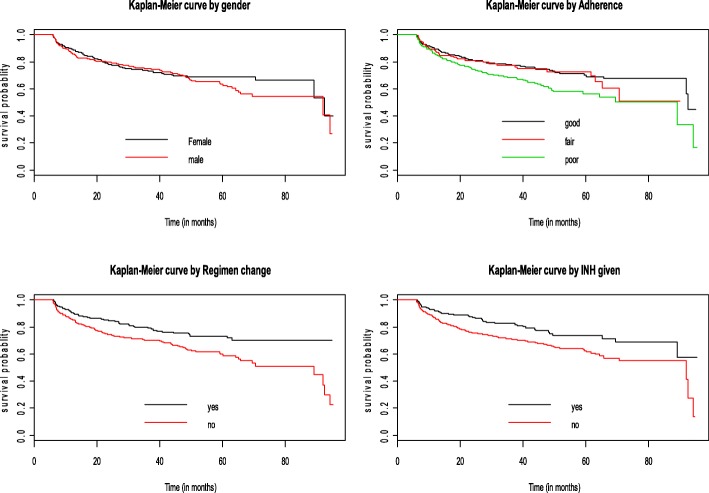
Kaplan-Meier survival estimator curve of second-line ART failure by predictor variables among adults in Amhara region (February 2008 – April 2016)

In the follow-up period, the median survival time of patients on second-line ART was 92 months. This implies that half of patients didn’t have treatment failure in the 92 months after starting second-line treatment (Fig. 1). The median survival times for male and female ART clients were 92.0 and 92.5 months respectively. Also, the median survival times for HIV positive patients who had poor ART adherence and good ART adherence were 89.2 and 92.5 months respectively. Furthermore, the median survival time was 89.2 months for those individuals who didn’t change second-line drug regimen at least once in the follow-up period. Moreover, the median survival time for clients who didn’t take INH preventive therapy was 92.0 months (Fig. 2)**.**

The median time to second-line treatment failure was 13.23 (IQR = 7.63, 25.50) months. The overall incidence rate of second-line treatment failure was 9.86 per 100 person-years in the study setting. In this study, treatment failure was high during the first and the last year of follow-up period (Table 3)**.**

**Table 3:** The incidence rates of second-line treatment failure in one year interval among adults living with HIV in Amhara Region (February 2008–April 2016)

One year interval	Person-time	failures	Incidence rate	95%confidence interval
1–12	10214.95	114	0.011	(0.009 0.013)
12–23	7286.56	68	0.009	(0.007 0.118)
23–34	5071.9	29	0.006	(0.004 0.008)
34–45	3420.44	18	0.005	(0.003 0.008)
45–56	2097	11	0.005	(0.003 0.009)
56–67	1168.18	8	0.007	(0.003 0.137)
67–78	442.43	2	0.005	(0.001 0. 018)
78–89	154.31	0	……	……
> 89	41.07	4	0.097	(0.036 0.259)
Total	30907.57	254	0.008	(0.007 0 .009)

### Predictors of second-line treatment failure

Modifying second-line drug regimen, ART adherence, WHO clinical stages at the switch, taking INH preventive therapy, and types of second-line drug regimen were found to be significant predictors of second-line treatment failure (Table 4)**.** The rate of treatment failure for patients who didn’t modify the second-line drug regimens (HR: 1.55, 95%CI: 1.18–2.04) was higher by 55% as compared to patients who modify the drug regimen at any time in the follow-up period. The rate of treatment failure was also higher by 40% for clients who had poor ART adherence (HR: 1.40, 95%CI: 1.06–1.85) as compared to those who had good ART adherence. The rate of second-line treatment failure was lower by 32% for those patients who were under WHO clinical stage III at switch (HR: 0.68, 95%CI: 0.50–0.91) as compared to patients who were under WHO clinical stage-I at switch. Furthermore, ART clients who were not taking INH preventive therapy (HR: 1.68, 95%CI: 1.23–2.30) were 1.68 times more likely to experience treatment failure as compared to patients who were taking INH preventive therapy. Clients who were under second-line regimen “TDF-3TC-LPV/r” (HR: 1.55, 95%CI: 1.03–2.32) were 1.55 times more likely to experience treatment failure than patients who were under second-line regimen “ABC-ddI-LPV/r”. Moreover, clients who were under second-line regimen “AZT-3TC-LPV/r” (HR: 3.00, 95%CI: 1.86–4.85) were three times more likely to experience treatment failure than patients who were under second-line regimen “ABC-ddI-LPV/r” (Table 4)**.**

**Table 4:** Univariable and multivariable semi-parametric proportional hazard model on predictors with time to second-line treatment failure among adults living with HIV in Amhara Region (February 2008–April 2016)

Variables	Crude hazard ratio (95%CI)	Adjusted hazard ratio(95%CI)
Age		
15–24	Reference	Reference
25–34	0.85 (0.56 1.29)	0.91 (0.60 1.39)
35–44	0.81 (0.52 1.25)	0.85 (0.54 1.32)
≥ 45	0.76 (0.45 1.28)	0.86 (0.50 1.47)
Gender		
Female	Reference	Reference
male	1.10 (0.86 1.41)	1.18 (0.91 1.53)
BMI(kg/m^2^)		
< 18.5	1.26 (0.98 1.62)	1.09 (0.83 1.42)
18.5–24.99	Reference	Reference
> 25	0.67 (0.39 1.20)	0.76 (0.44 1.33)
CD4 count at switch		
≥ 100 cells/mm^3^	Reference	Reference
< 100 cells/mm^3^	0.82 (0.64 1.05)	0.88 (0.68 1.14)
Regimen modification		
Yes	Reference	Reference
No	1.65 (1.27 2.14)	1.55 (1.18 2.04)
ART adherence		
good	Reference	Reference
Fair	1.11 (0.78 1.59)	1.12 (0.78 1.61)
poor	1.55 (1.18 2.03)	1.40 (1.06 1.85)
WHO clinical stage		
I	Reference	Reference
II	0.95 (0.59 1.53)	0.84 (0.52 1.39)
III	0.78 (0.59 1.04)	0.68 (0.50 0.91)
IV	1.44 (0.98 2.11)	1.14 (0.75 1.73)
Isoniazid Preventive Therapy (INH) given		
Yes	Reference	Reference
No	1.70 (1.25 2.32)	1.68 (1.23 2.30)
Functional status at switch		
Working	Reference	Reference
Ambulatory	1.45 (1.03 2.04)	1.42 (0.97 2.07)
bedridden	1.99 (0.98 4.05)	1.94 (0.93 4.05)
Opportunistic Infections (OI)		
Yes	Reference	Reference
No	1.00 (0.62 1.62)	0.98 (0.60 1.61)
Second-line regimen		
ABC-ddI-LPV/r	Reference	Reference
TDF-3TC-LPV/r	1.59 (1.08 2.35)	1.55 (1.03 2.32)
AZT-3TC-LPV/r	2.85(1.79 4.53)	3.00 (1.86 4.85)
Others^a^	1.89(1.28 2.82)	1.77 (1.19 2.67)

^a^ *= (ABC-ddI-NFV, AZT-3TC-ATV/r, TDF-3TC-ATV/r, ABC-3TC-LPV/r, and ABC-3TC-ATV/r)*

## Discussion

The main goal of this study was to estimate the incidence rate of second-line treatment failure and to identify its predictors among adults living with HIV in Amhara Region. The overall incidence rate of second-line treatment failure was 9.86 per 100 person-years. Semi-parametric proportional hazard model was performed and significant predictors of time to treatment failure were modification of the second-line drug regimen, ART adherence, WHO clinical stages, taking INH preventive therapy, and types of second-line drug regimen.

In this study, the median time to treatment failure was 13.23 (IQR = 7.63, 25.50) months. This result is in agreement with previous studies conducted in African and Asian countries [13, 18]. However, it was lower than a study conducted in western Kenya which was 37 months (IQR: 24–47) [19]. The possible explanations for this variation might be the differences in the study designs. The previous study employed case–control study design and it consider patients failing first-line ART as cases and patients who were not failing first-line ART as controls. As a result, computing the median failure time by merging cases and controls might give a higher value. The other possible reason might be the differences in the definition of treatment failure. The previous study used WHO 2006 immunological and clinical failure criteria, while our study used the WHO 2016 immunological and clinical failure criteria. The median time from second-line treatment failure was also lower for this study as compared to a study conducted in Rio de Janeiro, Brazil (40.0 months, 95% CI: 30.4–52.2) [20].

In the follow-up period, participants contributed a total of 2,575.63 person-years of observations, and the overall incidence rate of treatment failure was 9.86 (95%CI: 8.4–10.8) per 100 person years. The incidence rate of treatment failure for the current study was higher than previous studies conducted in northwest Ethiopia (61.7 per 1,000 person years) and southeast Ethiopia (9.38 per 1,000 person years) [16, 17]. The possible explanation of this difference might be the differences in the definition of treatment failure, because, previous studies used the WHO 2010 guideline, while our study used the WHO 2016 guideline. Also, the rate of second-line treatment failure was high during the first and the last year of follow-up period. This might be due to drug resistance is high in the first and the last year of follow-up [21]. In relation to this, second-line therapy has shown high early mortality but good virological suppression under programmatic conditions in India [22]. On the contrary, the rate of treatment failure on second-line therapy was low over the first year of follow-up in Johannesburg, South Africa [23].

The rate of treatment failure for clients who didn’t change the second-line drug regimens during resistance (HR: 1.55, 95%CI: 1.18–2.04) was higher as compared to patients who modify the drug regimen. The possible reasons for this result might be the changed regimen is more effective than previous drugs in reducing side effect and drug-drug interaction. The other possible reason might be the modified drug regimens could reconstitute CD4 cell count. The rate of second-line treatment failure for clients who had poor ART adherence (HR: 1.40, 95%CI: 1.06–1.85) was higher as compared to patients who had good ART adherence. It is reasonable that strict adherence to ART plays a crucial role in the success of therapy for peoples with HIV [24]. The rate of second-line treatment failure was decreased (HR: 0.68, 95%CI: 0.50–0.91) for patients who were under WHO clinical stage III as compared to clients who were on WHO clinical stage-I. Second-line treatment failure was higher (HR: 1.68, 95%CI: 1.23–2.30) for patients who were not taking INH preventive therapy as compared to patients who were taking it. It is reasonable that taking INH preventive therapy reduces the risk of tuberculosis and in turn it decreases the rate of treatment failure [25].

In the present study, Tenofovir (TDF) and Zidovudine (AZT) based second-line drug regimens have been shown to be associated more with treatment failure than Abacavir (ABC) based drug regimens. A study conducted in Kenya showed that patients on AZT based regimens had better performance of physical and mental health summary score compared to those on TDF [26]. On the other hand,a study conducted in southwest Ethiopia reported that TDF based drug regimens have shown better immunological recovery compared to AZT based regimens [27]. Finally, we recommend further studies to compare TDF, ABC and AZT based second-line drug regimens related to treatment efficacy.

### Limitation of the study

Though we did our best to estimate the incidence rate of second-line treatment failure and to identify its predictors, it is not free from limitations. The retrospective nature of the study limited the inclusion of all possible factors that could affect the incidence rate of treatment failure. Variables such as hemoglobin level and side effects were some of the plausible factors that were not measured in this study. Unavailability of viral load testing which is the gold standard method for treatment failure is also the limitation of the study. Since treatment failure in this study was monitored by CD4 cell count and WHO clinical stage, the incidence rate of treatment failure might be underestimated.

## Conclusions

In this study, the incidence rate of second-line treatment failure among adults living with HIV was high. Changing the second-line drug regimen, ART adherence, WHO clinical stages, taking INH, and types of second-line drug regimen were a significant predictors of time to treatment failure. Therefore, addressing predictor variables to prevent treatment failure among ART clients is essential and close monitoring to reduce the risk of treatment failure is also desirable.

## Data Availability

The datasets and materials used in this study are available upon request to the corresponding author.

## Abbreviations

3TC: Lamivudine
ABC: Abacavir
ART: Antiretroviral therapy
ARV: Antiretroviral
AZT: Zidovudine
ddI: Didanosine
HR: Hazard ratio
INH: Isoniazid
IQR: Inter quartile range
LPV/r: Lopinavir/ritonavir
OI: Opportunistic infections
TDF: Tenofovir
WHO: World Health Organization
